# Electroencephalographic Signatures of the Neural Representation of Speech during Selective Attention

**DOI:** 10.1523/ENEURO.0057-19.2019

**Published:** 2019-10-23

**Authors:** Vibha Viswanathan, Hari M. Bharadwaj, Barbara G. Shinn-Cunningham

**Affiliations:** 1 Weldon School of Biomedical Engineering, Purdue University, West Lafayette, IN 47907; 2 Department of Speech, Language, and Hearing Sciences, Purdue University, West Lafayette, IN 47907; 3 Neuroscience Institute, Carnegie Mellon University, Pittsburgh, PA 15213

**Keywords:** cocktail-party problem, EEG, gamma rhythms, selective attention, speech coding, theta rhythms

## Abstract

The ability to selectively attend to speech in the presence of other competing talkers is critical for everyday communication; yet the neural mechanisms facilitating this process are poorly understood. Here, we use electroencephalography (EEG) to study how a mixture of two speech streams is represented in the brain as subjects attend to one stream or the other. To characterize the speech-EEG relationships and how they are modulated by attention, we estimate the statistical association between each canonical EEG frequency band (delta, theta, alpha, beta, low-gamma, and high-gamma) and the envelope of each of ten different frequency bands in the input speech. Consistent with previous literature, we find that low-frequency (delta and theta) bands show greater speech-EEG coherence when the speech stream is attended compared to when it is ignored. We also find that the envelope of the low-gamma band shows a similar attention effect, a result not previously reported with EEG. This is consistent with the prevailing theory that neural dynamics in the gamma range are important for attention-dependent routing of information in cortical circuits. In addition, we also find that the greatest attention-dependent increases in speech-EEG coherence are seen in the mid-frequency acoustic bands (0.5–3 kHz) of input speech and the temporal-parietal EEG sensors. Finally, we find individual differences in the following: (1) the specific set of speech-EEG associations that are the strongest, (2) the EEG and speech features that are the most informative about attentional focus, and (3) the overall magnitude of attentional enhancement of speech-EEG coherence.

## Significance Statement

Difficulty understanding speech amid competing talkers is the most common audiological complaint. However, the brain mechanisms that support our ability to selectively attend to a target speech source in a mixture are poorly understood. Here, we use electroencephalography (EEG) to systematically map the relationships between features of input speech and those of neural responses, when speech is attended versus ignored. We show that EEG rhythms in different canonical frequency bands, including the γ band, preferentially track fluctuations in attended speech over ignored speech. However, the strength and pattern of attention effects also show individual differences. These results can inform computational models of selective attention and assistive listening devices such as EEG-guided hearing aids.

## Introduction

Most of us take for granted our ability to understand speech amid the cacophony we encounter every day (Cherry, 1953), an ability that is unparalleled by machine algorithms (Loizou, 2013). However, 3–5% of children and approximately one in five adults find communicating in noisy social situations extremely challenging (Chermak and Musiek, 1997; Lin et al., 2011), including some listeners who have clinically normal or near-normal thresholds (Kumar et al., 2007). The brain mechanisms that support this auditory “selective attention” process are poorly understood. Identifying correlates of how speech is represented in the brain during selective attention would give us insight into the mechanisms of this process, and how it fails in different clinical populations. Here, we use electroencephalography (EEG) to probe how attended and ignored speech streams in a sound mixture are represented in the brain. Specifically, our goal was to characterize which acoustic features of the speech streams are related to which features of the EEG response, and how such relationships differ for attended and ignored streams.

Neurophysiological experiments using EEG and MEG (magnetoencephalography) show that brain rhythms are intimately associated with sensory processing (Buzsáki and Draguhn, 2004). Electrophysiological studies and computational models suggest that gamma rhythms (30–90 Hz) support the formation of cell assemblies (Cannon et al., 2014). Such assemblies likely mediate stimulus competition and attentional selection of task-relevant representations (Börgers et al., 2008). In contrast, delta (1–3 Hz) and theta (3–7 Hz) oscillations may reflect synchronous interactions between assemblies (White et al., 2000). Strikingly, speech also has spectro-temporal features that are quasiperiodic over similar time scales. Perceptually, the energy envelopes of different frequencies spanning the hearing range carry important information about speech content (Shannon et al., 1995; Elliott and Theunissen, 2009). Importantly, the time scales of phonemic, syllabic, and phrase/sentence level rhythmic fluctuations in speech parallel the EEG gamma, theta, and delta, frequencies, respectively. This has led researchers to speculate that the canonical cortical network oscillations are involved in the processing of speech sounds (Giraud and Poeppel, 2012; Doelling et al., 2014). For speech in isolation, brain oscillations phase lock to the speech fluctuations, or more precisely, the fluctuations conveyed at the output of cochlear processing of speech sounds (Ghitza et al., 2012; Gross et al., 2013). It has been suggested that the temporal match between inherent cortical network oscillations and the natural fluctuations in communication sounds may help the listener parse input speech (Luo and Poeppel, 2007; Ghitza and Greenberg, 2009; Gross et al., 2013).

Fundamental to our understanding of everyday communication is the question of how the neural computations generating brain oscillations relate to the perceptual processes of scene segregation and attentional selection (Shinn-Cunningham, 2008). EEG/MEG studies show that when a mixture of speech sources is presented, low-frequency cortical responses (matching canonical delta and theta bands) preferentially track the temporal envelopes of attended speech compared to simultaneously presented ignored speech (Ding and Simon, 2012; O’Sullivan et al., 2015). Similarly, electrocorticography (ECoG) studies show that the power of brain oscillations in the high-gamma (70–150 Hz) band preferentially phase locks to attended speech more than ignored speech (Mesgarani and Chang, 2012; Golumbic et al., 2013). While non-invasive studies have focused on low-frequency portions of the EEG, invasive studies have focused on the high-frequency bands. To the best of our knowledge, no non-invasive studies to date have reported how the full complement of canonical brain oscillations track speech sounds in a mixture of competing sources, when attention is selectively directed to one source stream.

Here, we systematically study how brain oscillations in each of the canonical frequency bands are related to speech fluctuations, comparing when the speech is attended versus when it is ignored. Specifically, we analyze EEG data recorded during a realistic selective attention task, and replicate previous findings that low-frequency EEG bands (in the delta and theta range) show enhanced synchrony with a speech stream when it is attended compared to when it is ignored. In addition, we find that the envelope of the low-gamma EEG band also shows enhanced synchrony with the target speech. Finally, we observe individual differences in the strength and pattern of attention effects. We discuss the implications of our findings for basic neuroscience, and their potential for informing brain-computer interface (BCI) applications such as EEG-guided hearing aids (Fuglsang et al., 2017; Fiedler et al., 2017; O’Sullivan et al., 2017; Van Eyndhoven et al., 2017).

## Materials and Methods

### Participants

Data were collected from twelve human subjects (six female), aged 23–41 years, recruited from the Boston University community. All subjects had pure-tone hearing thresholds better than 20-dB hearing level (HL) in both ears at standard audiometric frequencies between 250 Hz and 8 kHz. Subjects provided informed consent in accordance with protocols established at Boston University. Of the twelve subjects who participated, data from two were excluded from analysis for reasons described below.

### Experimental design

In each listening block, two running speech streams (narrated whole stories), one spoken by a male and the other by a female (from one of “The Moth” storytelling events, New York), were presented simultaneously to the subject. The stories were each lateralized using interaural time delays (ITDs). The root-mean-square intensities of the male and female speech streams were equalized dynamically using a sliding window of length 2 s. A total of four stories were used in the experiment. Each subject performed four blocks; at the beginning of each block, subjects were verbally instructed to attend to one of the two talkers throughout that block. Subjects were also asked to stay still with their eyes blinking naturally during the experiment; however, their eye gaze was not restricted. EEG was measured simultaneously with the behavioral task in each block. The individual stories were ∼9–12 min long; thus, the blocks were also 9–12 min long each.

At the end of each block, subjects were given a quiz on the attended story. If a subject answered at least 90% of the quiz questions correctly, they passed the quiz. Based on the responses to the quiz, one subject was excluded due to their inability to accurately recall details of the attended story. All of the remaining eleven subjects were able to recount details of the attended story accurately, and reported being largely unaware of the details of the other (ignored) story.

All the subjects were presented with the same set of speech stories. However, which story was attended in a given block was varied randomly across listeners, with the constraint that each listener heard every story once when it was to be ignored and once when it was to be attended. This design allowed us to directly compare attended and ignored conditions for the same acoustic input to the subject. Furthermore, the two presentations of each speech story (once when the story was to be attended, and the other when it was to be ignored) were separated by at least one block for every subject.

### Data acquisition

A personal desktop computer controlled all aspects of the experiment, including triggering sound delivery and storing data. Special-purpose sound-control hardware (System 3 real-time signal processing system, including digital-to-analog conversion and amplification; Tucker Davis Technologies) presented audio through insert earphones (ER-1; Etymotic) coupled to foam ear tips. The earphones were custom shielded using a combination of metallic tape and metal techflex to attenuate electromagnetic artifacts. The absence of measurable electromagnetic artifact was verified by running intense click stimuli through the transducers with the transducers positioned in the same location relative to the EEG cap as actual measurements, but with foam tips left outside the ear. All audio signals were digitized at a sampling rate of 24.414 kHz. The EEG signals were recorded at a sampling rate of 2.048 kHz using a BioSemi ActiveTwo system. Recordings were done with 32 cephalic electrodes, additional electrodes on the earlobes, and a bipolar pair of electrodes adjacent to the outer left and right canthi to measure saccadic eye movements.

### Data preprocessing

The EEG signals were re-referenced to the average of all the channels. The signal-space projection method was used to construct spatial filters to remove eye blink and saccade artifacts (Uusitalo and Ilmoniemi, 1997). The broadband EEG was then bandpass filtered between 1 and 120 Hz for further analysis. For computing associations between speech and EEG, the EEG data were segmented into 5-s-long epochs. Epochs with movement artifacts were identified as those with a peak-to-peak swing that exceeded twenty median absolute deviations compared to the median epoch. All such epochs were rejected to eliminate movement artifacts. Of the eleven subjects who successfully passed our behavioral screening, one subject was excluded because >20% of their EEG data were contaminated by movement artifacts. The data from the remaining ten subjects were used in all further analyses.

### Estimating speech-EEG associations

Our goal was to understand the relationships between features of input speech and EEG responses, and how these relationships vary depending on whether speech is attended to or ignored. For the speech features, we considered envelope fluctuations in ten different frequency bands. For the EEG features, we considered different EEG bands corresponding to the canonical cortical rhythms, and different scalp locations of the 32-channel EEG recording. The rationale for the choice of these speech and EEG features, along with the procedure for extracting them are described below.

The auditory periphery can be approximated as a filter bank that decomposes speech into different frequency bands; the envelope at the output of each cochlear filter is conveyed to the brain by auditory-nerve fibers tuned to the corresponding frequency band (Khanna and Leonard, 1982; Smith et al., 2002). We used a bank of ten gammatone filters that mimic cochlear frequency selectivity (Slaney, 1993), with center frequencies spanning 100–8533 Hz. The filters were spaced roughly logarithmically, such that their center frequencies had best places that are spaced uniformly along the length of the cochlea according to an established place-frequency map (Greenwood, 1990). The amplitude envelope at the output of each filter, extracted using the Hilbert transform, was treated as a distinct speech feature. For the speech signals used in our experiment, the envelopes at the different filters were not strongly correlated. In analyzing the speech envelopes extracted from different bands, we found that the variance explained in the envelope of one band by any other band was ∼8% or less (estimated by calculating squared coherence between speech envelopes). This suggests that the speech envelopes in the ten different cochlear bands provide somewhat complementary speech information.

Previous EEG/MEG studies show that cortical responses to speech mixtures preferentially track the spectro-temporal features of the attended speech during selective listening (Ding and Simon, 2012; O’Sullivan et al., 2015). Specifically, the low-frequency speech envelope elicits phase-locked EEG responses at corresponding frequencies (delta band: 1–3 Hz, and theta band: 3–7 Hz). Furthermore, ECoG studies show that the slowly varying envelopes of high-frequency neural responses (high-gamma band: >70 Hz) also track the attended speech (Mesgarani and Chang, 2012; Golumbic et al., 2013). Thus, we systematically studied the relationship between speech and the corresponding neural responses by decomposing the EEG signal from each of the 32 channels into six canonical frequency bands (delta: 1–3 Hz, theta: 3–7 Hz, alpha: 7–15 Hz, beta: 13–30 Hz, low-gamma: 30–70 Hz, and high-gamma: 70–120 Hz; Buzsáki and Draguhn, 2004). In the delta, theta, alpha, and beta bands, the filtered EEG signal was treated as a feature. On the other hand, for the higher-frequency gamma bands, we were motivated by the results from the ECoG studies to extract and use the amplitude envelopes in those bands instead (discarding phase information). For the alpha and beta bands, we considered the amplitude envelopes of those bands as additional features separately from the filtered EEG. This choice was motivated by the finding that alpha power fluctuates coherently with the attended stimulus (Wöstmann et al., 2016), and that beta-band power fluctuates in a task-specific way across many cognitive and motor tasks (Engel and Fries, 2010). To extract the envelopes of the alpha, beta, low-gamma, and high-gamma bands, we used the Hilbert transform. Overall, a total of 256 EEG features were considered: the filtered EEG in the delta, theta, alpha, and beta bands, and the envelopes of alpha, beta, low-gamma, and high-gamma bands, across the 32 EEG channels. Throughout this report, we will use the term EEG bands to denote the EEG signals or envelope signals in different frequency bands. Thus, the analyzed EEG bands consist of the delta, theta, alpha, and beta bands, and the amplitude envelopes of alpha, beta, low-gamma, and high-gamma bands.

Spectral coherence (also simply referred to as coherence) was chosen as the measure of statistical dependence between the speech and EEG signals. High coherence indicates a consistent phase relationship between signals (Hannan, 1970; Thomson, 1982; Dobie and Wilson, 1989). Moreover, when artifactual trials are excluded, spectral coherence is likely to be more sensitive than the phase-locking value (Lachaux et al., 1999), as coherence computation assigns greater weights to trials with larger signal amplitude (Dobie and Wilson, 1994). A multi-taper approach (with five tapers, resulting in a frequency resolution of 1.2 Hz) was used to estimate the spectral coherence between each speech and EEG feature from the 5-s-long epochs segmented from the raw EEG data (Slepian, 1978; Thomson, 1982). A total of 108 epochs were used in the computation of each coherence spectrum. The multi-taper estimate minimizes spectral leakage (i.e., reduces mixing of information between far-away frequencies) for any given spectral resolution, and is calculated from the Fourier representations of two signals *X*(*f*) and *Y*(*f*) as follows:

(1) $$C_{XY}(f)=\frac{S_{XY}(f)}{\sqrt{S_{XX}(f)S_{YY}(f)}},$$

where

(2) $$S_{XY}(f)=\frac{1}{K_{tapers}N_{epochs}}\sum_{k=1}^{K_{tapers}}\left|\sum_{n=1}^{N_{epochs}}X_{kn}(f)Y_{kn}^{*}(f)\right|,$$

(3) $$S_{XX}(f)=\frac{1}{K_{tapers}N_{epochs}}\sum_{k=1}^{K_{tapers}}\left|\sum_{n=1}^{N_{epochs}}X_{kn}(f)X_{kn}^{*}(f)\right|,$$

(4) $$S_{YY}(f)=\frac{1}{K_{tapers}N_{epochs}}\sum_{k=1}^{K_{tapers}}\left|\sum_{n=1}^{N_{epochs}}Y_{kn}(f)Y_{kn}^{*}(f)\right|.$$

**Figure 1.**
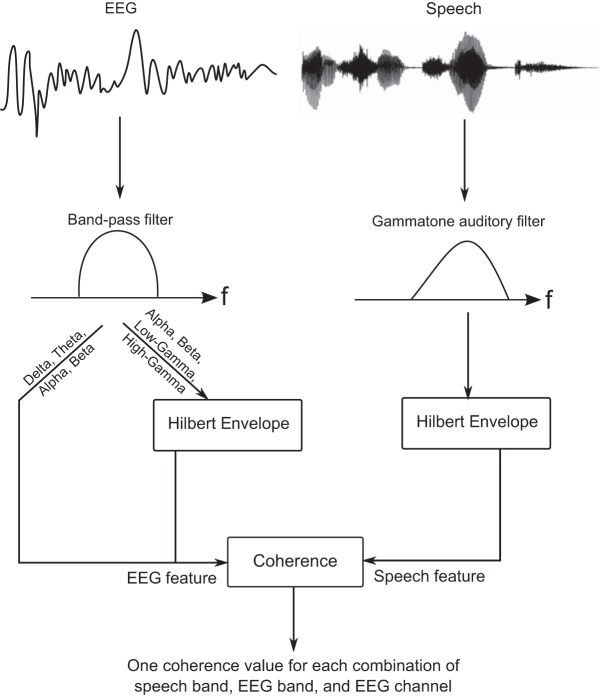
Illustration of the steps used to extract speech and EEG features and to estimate the association between them. The speech signal is passed through a gammatone filter bank simulating cochlear processing, and the envelope at the output of each filter (i.e., the envelope of each speech band) is extracted as a speech feature. Similarly, different bands of the EEG and different sensor channels together form the different EEG features. For the lower-frequency bands (delta and theta), the EEG signals are used as is. For the alpha and beta bands, both the signals in those bands, and their envelopes are extracted as separate features. For the higher-frequency gamma bands, only the envelopes of the EEG signals in those bands are considered. These EEG features are then compared with the speech features using spectral coherence.

For each pair of speech and EEG features, a single measure of coherence was obtained by averaging the coherence spectrum obtained via the multi-taper estimation procedure as follows: For the regular coherence in the delta, theta, alpha, and beta bands, the coherence values were averaged over the canonical frequency ranges of the respective bands (i.e., 1–3 Hz for delta, 3–7 Hz for theta, 7–15 Hz for alpha, and 13–30 Hz for beta). For the envelope coherences of the alpha, beta, low-gamma, and high-gamma bands, the averaging was performed over envelope frequencies of 1–7 Hz (corresponding to the frequency range at which previous studies report phase locking between the speech envelope and the envelope of the neural response in the gamma band; Gross et al., 2013). Figure 1 summarizes the steps used to extract speech and EEG features, and to estimate the coherence between them.

In this way, we characterized the relationships between different features of input speech (i.e., the speech envelopes in different cochlear bands) and different features of the EEG response (each of which corresponds to a specific EEG band and channel). In particular, we characterized these relationships in an attention-specific manner, i.e., both when the input speech was attended and also when it was ignored. This allowed us to examine the effects of attention on the speech-EEG relationships separately in different EEG bands, different scalp locations, and different speech bands, and also to characterize individual differences in the attentional enhancement of speech-EEG associations. Further methodological details are presented alongside each result description as needed.

### Visualizing individual subject results as a network graph

The full set of speech-EEG relationships is a high-dimensional data set (with EEG bands, scalp channels, and speech bands constituting the different dimensions) that can be conceived of as a network. In many domains, bipartite graphs have been successfully used to represent and characterize the complex pattern of associations between two types of variables (“nodes”) in a relational network [e.g., group-member relationships in a social network (Wilson, 1982), genotype-phenotype relationships in a biological network (Goh and Choi, 2012), etc.]. To visualize the relationships between all pairs of speech and EEG features simultaneously in each individual subject, we constructed bipartite graphs with the ten speech features forming the nodes in one partition, and the 256 EEG features (32 scalp locations × eight EEG bands) forming the nodes in the other. An edge (i.e., connection) between a speech feature and an EEG feature in our bipartite graph construction signifies a statistical dependence between them, such as a significant coherence value. We constructed separate attended and ignored graphs for each individual subject in our study using the following procedure. First, the speech-EEG coherences for each subject were averaged across all speech stories for the attended and ignored conditions separately. Next, edges were drawn between those pairs of speech-EEG features whose coherence values met a particular threshold. The resulting graph representations of speech-EEG relationships were visualized to qualitatively compare the two attention conditions and different individuals. To quantitatively compare attended and ignored graphs, we computed the average difference in the number of graph edges between the attended and ignored conditions, for different coherence thresholds. The results were compared with permutation-based null distributions to obtain *p* values, as described in Statistical analysis.

The bipartite graph formulation also has the advantage that the complex set of dependencies between speech and EEG, and how those dependencies are modulated by attention, can be summarized using rigorous metrics developed in network science. Accordingly, we take advantage of network summary measures that use the entire network structure to find those speech and EEG features that best capture attentional focus in an individual-specific manner. This is done with the view of informing attention-decoding applications as to which EEG and stimulus features may provide the best decoding performance at the individual level. For this, we first computed the differential (“attended–ignored”) coherence for each speech-EEG pair for each individual subject (but averaged across speech stories). For each individual, the full set of speech and EEG features and their associated differential coherences can be represented as a weighted “differential” speech-EEG bipartite graph, with the differential coherence associated with each speech-EEG pair forming the edge weight for that pair. Note that this weighted graph representation of the differential coherences contrasts with the unweighted graph representations for the attended and ignored conditions that were described previously. For the attended and ignored graphs, we had used a coherence threshold to define an edge. On the other hand, to obtain the differential graphs, we did not use any thresholding procedure. Instead, the differential coherence values across all speech-EEG feature pairs were retained, and used to define graph edge weights. Finally, to find those speech and EEG features that are the most informative about an individual’s attentional focus, we computed the eigenvector-based graph centrality measure for each speech and EEG feature in every individual’s differential graph. For a discussion on the notion of network centrality, and how it may be computed in bipartite graphs to identify the most informative nodes in the network, see Faust (1997).

### Statistical analysis

The primary question that this study is concerned with is whether the neural representation of speech is modulated by attention. For this, the null hypothesis is that attention does not alter speech-EEG relationships. We used a non-parametric within-subjects randomization procedure to perform statistical inference against this null hypothesis. This procedure was applied to two separate analyses, as described below.

For the analysis performed to characterize which EEG bands show attention-dependent changes in coherence with speech (results in Fig. 3*A*), the specific null is that the speech-EEG coherence in each of the EEG bands is the same on average for the attended and ignored conditions. Thus, under the null hypothesis, the attended and ignored conditions are equivalent and the labels “attended” and “ignored” can be swapped randomly to generate examples of coherence differences that would be observed under the null hypothesis. Note that our experimental design of randomly assigning which of the two stories in each block is attended provides the necessary exchangeability criterion, justifying the permutation procedure (Nichols and Holmes, 2002). That is, every permutation of the order in which the stimuli and attention conditions occurred was equally likely to occur during data acquisition. Thus, under the null hypothesis, the condition labels corresponding to the measurements can be randomly permuted. To generate a single realization from the null distribution, a random sign was assigned to the coherence difference between the attended and ignored conditions for each subject and speech story, then the results were averaged across subjects and stories. This procedure was repeated with 500,000 distinct randomizations to generate the full null distribution for the average coherence difference. A separate null distribution was generated for each of the eight EEG bands using band-specific data. For each band, the corresponding null distribution was used to assign a *p* value to the observed average coherence difference obtained with the correct labels. Finally, to correct for multiple comparisons across the eight EEG bands, the conservative Bonferroni procedure was used. In addition to being used to obtain *p* values, the null distributions were also used to express each individual’s coherence-difference values as a *z*-score, which provided an easy-to-interpret quantification of effect sizes. We used a similar permutation procedure to generate noise floors for computing the *z*-scores shown in Figure 3*B*,*C*, and in the differential scalp map of Figure 4. A separate noise floor was generated for each speech band in Figure 3*B*, for each pixel (corresponding to a distinct speech band and EEG band) in Figure 3*C*, and for each electrode in Figure 4.

For the analysis on the number of edges in the graph representation of speech-EEG coherence (Fig. 7), a similar permutation procedure was used. Here, the specific null hypothesis is that the graph has the same number of edges in the attended and ignored conditions on average. Thus, for each subject, a random sign was assigned to the difference in the number of edges between the attended and ignored conditions, then the result was averaged over subjects. This randomization procedure was repeated 500,000 times to generate the full null distribution. A separate null distribution was generated for each of the coherence thresholds shown in Figure 7. The observed average differences in the number of edges between the correctly labeled attended and ignored conditions were then compared to the corresponding null distributions to assign *p* values.

The noise floor parameters used for computing the *z*-scores shown in the attended and ignored scalp maps of Figure 4 were theoretically derived. This was done by using the mean and variance expressions for multi-taper coherence estimates provided in Bokil et al. (2007), and adjusting the variance parameter to account for pooling across EEG frequencies and speech bands.

### Software accessibility

Stimulus presentation was controlled using custom MATLAB (The MathWorks, Inc.) routines. EEG data preprocessing was performed using the open-source software tools MNE-Python (Gramfort et al., 2013, 2014) and SNAPsoftware (Bharadwaj, 2018). All further analyses were performed using custom software in Python (Python Software Foundation; www.python.org). Network visualizations were created using the SAND package (Kolaczyk and Csárdi, 2014) in R (R Core Team; www.R-project.org). Copies of all custom code can be obtained from the authors.

## Results

**Figure 2.**
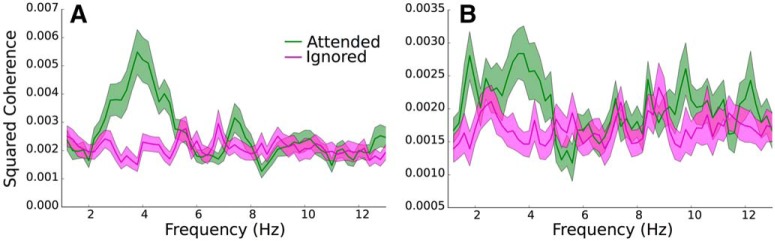
Illustration of the effect of attention on the average speech-EEG magnitude squared coherence spectra, for (***A***) the envelope of the 1014-Hz speech band, and the low-frequency portions (overlapping with the delta and theta bands) of EEG channel C3, and for (***B***) the envelope of the 3733-Hz speech band, and the envelope of the low-gamma band of EEG channel CP1. Note that the *y*-axis ranges differ between ***A*** and ***B***. The shaded regions indicate values within 1 SEM. The delta-band and theta-band EEG responses (***A***), and the low-gamma-band EEG envelope fluctuations (***B***), selectively track features of the attended speech over the ignored speech.

Figure 2 shows magnitude squared coherence spectra (averaged over subjects and speech stories) for two example speech-EEG pairings: the envelope of the 1014-Hz speech band and the low-frequency EEG in sensor C3 (Fig. 2*A*), and the envelope of the 3733-Hz speech band and the envelope of the low-gamma EEG band in sensor CP1 (Fig. 2*B*). The coherence in the attended condition is greater than that in the ignored condition in the 2- to 5-Hz frequency range (overlapping with the delta and theta bands) for the example in Figure 2*A*. The slow envelopes of the low-gamma band also preferentially track attended speech in the 2- to 5-Hz frequency range (Fig. 2*B*).

**Figure 3.**
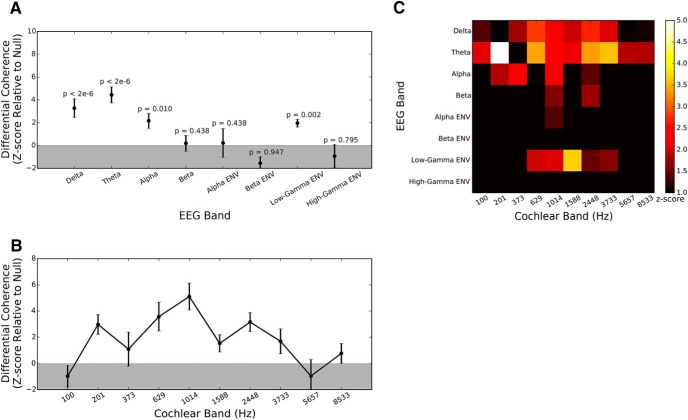
Differential effects of attention on speech-EEG coherences in different EEG bands (***A***), different speech bands (***B***), and the full matrix of EEG bands versus speech bands (***C***). ***A***, Differential (attended–ignored) coherence averaged across speech bands and EEG channels (shown as a *z*-score) for each of the EEG bands. Uncorrected *p* values obtained from the permutation test are displayed for the different EEG bands. When a Bonferroni-corrected *p* value threshold of 0.05/8 = 0.006 is applied to each band, we find that the delta and theta bands show significantly higher coherence with speech when it is attended compared to when it is ignored. In addition, we also find that the envelope of the low-gamma band shows greater coherence with attended versus ignored speech. ***B***, Differential coherence averaged across all EEG bands and EEG channels (shown as a *z*-score) for each input speech band. The strongest attention effects appear to occur in the 0.5- to 3-kHz range, which contains spectro-temporal speech features (formants and formant transitions) that convey many vowel and certain consonant cues, and is also the range thought to be the most important for speech intelligibility. In panel ***C***, the differential coherence averaged across EEG channels is shown as a *z*-score for each EEG band and speech band for completeness. While the 0.5- to 3-kHz speech frequency range shows hot spots in the delta, theta, and low-gamma EEG bands, the lower-frequency speech bands (e.g., 200 Hz) show a hot spot only in the theta range corresponding to the syllabic rate. This could be because the pitch conveyed by the resolved harmonics of the syllabic voicing may be an important cue based on which attention is directed. In all three panels, *z*-scores shown are averaged across speech stories and individual subjects, with error bars representing the SE.

As described above in Materials and Methods, Estimating speech-EEG associations, the coherence spectrum for each pair of speech-EEG features was averaged across frequencies to obtain a single coherence value for that feature pair; this was done separately for the attended and ignored conditions. One key question we wished to answer was which EEG bands showed the greatest attention effects. To address this question, we averaged the differential coherences (attended–ignored) for each EEG band across all speech bands and across the 32 EEG channels. The results obtained from this analysis are shown in Figure 3*A*. For each EEG band, we statistically tested whether the coherence increase in the attended condition was significant using the permutation procedure described previously. To correct for multiple comparisons across the eight EEG bands that were considered, we used a Bonferroni correction with a familywise error rate of 0.05. Thus, for each of the eight tests, only *p* < 0.05/8 were considered to be statistically significant. Based on the statistical tests, we find that both delta and theta bands of the EEG show greater coherence with a speech stream when that stream is attended compared to when it is ignored (i.e., a positive attended–ignored difference). This replicates previously reported results (Ding and Simon, 2012; O’Sullivan et al., 2015). Aside from the attention-dependent increase in low-frequency coherence, we also observe that the envelope of the low-gamma band shows greater coherence to speech in the attended condition. The preferential synchrony of gamma-band envelopes with attended speech has previously been reported only in invasive recordings (Mesgarani and Chang, 2012; Golumbic et al., 2013). For speech in isolation, some non-invasive studies have found gamma-band envelopes to be synchronous with input speech (Gross et al., 2013); however, to the best of our knowledge an attention-dependent increase of this coherence has previously not been reported with non-invasive recordings.

In addition to identifying the EEG bands that showed the greatest attention effects, we were also interested in characterizing which speech bands contribute most to attention-dependent increases in coherence. To address this question, we averaged the differential coherences for each speech band across the 32 scalp locations and across all EEG bands. This yielded a profile of attention-dependent increases in coherence across the ten different speech bands. The results are shown in Figure 3*B*. The strongest attention effects appear to occur in the 0.5- to 3-kHz range, which contains spectro-temporal speech features (formants and formant transitions) that convey many vowel and certain consonant cues (Gold and Morgan, 2002), and is also the range thought to be the most important for speech intelligibility (Kryter, 1962).

To examine whether the attention effects for different speech bands varied with the EEG bands that they were paired with, we visualized the differential coherence for the full matrix of speech bands versus EEG bands, averaged across EEG channels. The results are shown in Figure 3*C*. While the 0.5- to 3-kHz speech frequency range shows hot spots in the delta, theta, and low-gamma EEG bands, the lower-frequency speech bands (e.g., 200 Hz) show a hot spot only in the theta range corresponding to the syllabic rate. This could be because the pitch conveyed by the resolved harmonics of the syllabic voicing may be an important cue based on which attention is directed.

**Figure 4.**
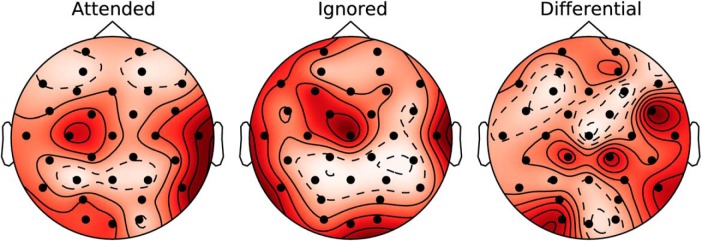
Scalp maps showing the average coherence (shown as a *z*-score) at each of the different EEG electrodes in the attended, ignored, and differential conditions. To obtain the scalp maps, the speech-EEG coherence values were averaged across the delta, theta, and low-gamma EEG bands (i.e., the bands showing significant attention effects in Fig. 3*A*), and all speech bands, and expressed as a *z*-score. The intensity shown at each electrode is the mean of the *z*-score across speech stories and individual subjects. Note that the scalp maps are scaled to their respective minimum and maximum *z*-score values, so as to best show the spatial patterns. The spatial profiles are hard to distinguish between the attended and ignored maps; however, note that the coherences are larger in the attended condition than the ignored, on an absolute scale. The differential map shown in the right column quantifies these differences across the scalp. Temporal-parietal regions appear to show the largest coherence differences between the attended and ignored conditions; however, this pattern is not symmetric between the hemispheres.

We also wished to find the EEG scalp locations that show the greatest coherence and attention effects. To address this question, we averaged the coherence values across the ten speech bands, and the delta, theta, and low-gamma EEG bands (i.e., the bands showing significant attention effects in Fig. 3*A*). The results are plotted as a topographic map of coherence values (i.e., one value for each of the 32 scalp locations) for the attended, ignored, and differential conditions, respectively, in Figure 4. The spatial profiles are hard to distinguish between the attended and ignored maps; however, note that the coherences are larger in the attended condition than the ignored, on an absolute scale. The differential map quantifies these differences across the scalp. Temporal-parietal regions appear to show the largest coherence differences between the attended and ignored conditions; however, this pattern is not symmetric between the hemispheres. This result is consistent with previous studies that found that areas such as the superior temporal gyrus and the inferior parietal lobule contribute to attention effects (Golumbic et al., 2013). In addition to plotting scalp maps averaged across EEG bands, we also looked at band-specific scalp maps for the differential condition. However, the spatial patterns in those maps were not easily interpretable, and are hence not shown here. Because we only used 32 channels, a detailed exploration of which brain sources contribute to the observed differential coherences cannot be done with our data. This should be a focus of future studies.

**Figure 5.**
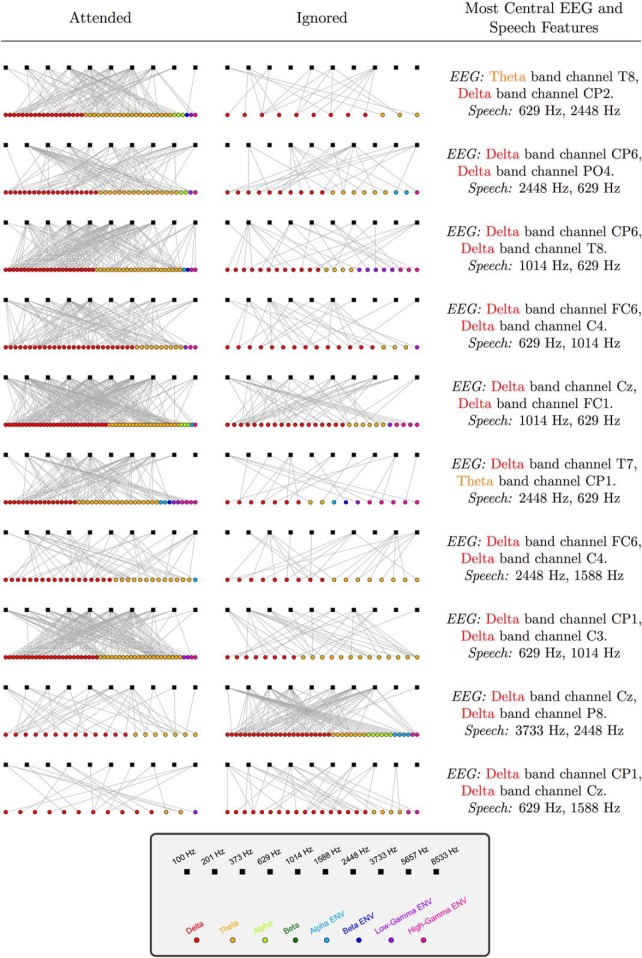
Graph representation of speech-EEG coherence in the attended and ignored conditions for all individual subjects. Rows represent different individuals. Squares denote speech features (i.e., the envelopes from the ten speech bands; shown in the order of increasing center frequency). Each circle denotes an EEG feature (i.e., a particular EEG band from a particular scalp location). An edge between a speech and EEG feature indicates that the coherence between them meets a threshold of 3 SDs from the mean. Only EEG features with one or more edges that survive the thresholding procedure are shown. Attended graphs exhibit greater number of edges compared to ignored graphs for all but two subjects (see bottom two rows). Additionally, the graph structure is variable across subjects. The top two EEG and speech features that are most informative (as obtained using eigenvector centrality) about an individual’s attentional focus also vary across subjects (rightmost column).

The results shown so far were mainly concerned with attention-dependent coherences averaged across different sets of speech and EEG features (i.e., across speech bands, and/or EEG bands, and/or scalp locations). In addition to this, we also constructed speech-EEG bipartite graphs for each individual to examine the full set of coherence values corresponding to all pairs of speech-EEG features simultaneously. Figure 5 shows attended and ignored graphs (averaged over speech stories) for all individual subjects in our study. In this figure, each square denotes a speech feature, and each circle denotes an EEG feature. An edge is shown connecting a pair of speech-EEG features if the coherence between them meets a certain threshold. Here, a coherence threshold of 3 SDs from the average coherence (pooled across attended and ignored conditions) is arbitrarily chosen, and only edges whose coherence meets that threshold are shown. One pattern that is immediately apparent from Figure 5 is that there are many more edges in the attended condition than in the ignored condition for eight of the ten subjects in this study. This suggests that a larger number of speech-EEG feature pairs become coherent when the speech is attended. Also apparent from Figure 5 is the fact that the graph structure is variable across subjects. This means that the particular speech-EEG feature pairs that show the greatest coherence values are not the same across subjects. As described above in Materials and Methods, Visualizing individual subject results as a network graph, we used the eigenvector centrality measure for bipartite graphs to find those EEG and speech features that are the most informative about an individual’s attentional focus. We find that the most central features differ between individuals, as shown in Figure 5. This suggests that for applications such as BCIs that aim to decode attention from EEG, individual-specific customization of features might be necessary to obtain optimal decoding performance.

**Figure 6.**
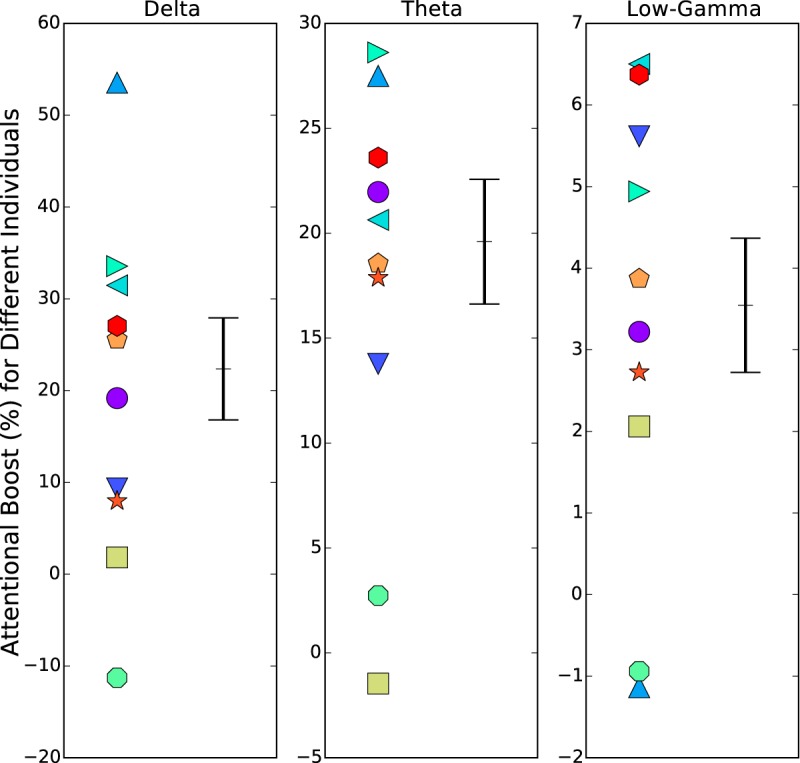
Individual differences in the overall magnitude of attentional enhancement of speech-EEG coherences in different EEG bands. Each individual’s attentional boost in coherence is shown (with an individual-specific marker symbol and color) for the delta, theta, and low-gamma EEG bands (i.e., the bands showing significant attention effects in Fig. 3*A*). The mean and SE across individuals are also indicated in black. Note that the *y*-axis ranges differ between the three panels of the figure. The attentional boost was computed as the percentage change in squared coherence going from the ignored condition to the attended, averaged across EEG channels, speech bands, and the different speech stories. The distribution of the attentional boost across individuals is skewed above zero in all three EEG bands, consistent with positive attentional boost in the neural coding of target speech. Furthermore, there is considerable variation across subjects almost uniformly over the range of boosts.

Figure 6 shows individual differences in the overall magnitude of attentional enhancement of speech-EEG coherences, separately for the delta, theta, and low-gamma EEG bands (i.e., the bands showing significant attention effects in Fig. 3*A*). Here, each individual’s “attentional boost” was computed as their percentage change in squared coherence going from the ignored condition to the attended, averaged across the 32 EEG channels, all speech bands, and the different speech stories. This attentional boost metric represents the percentage change in the proportion of EEG signal energy that is correlated with a speech signal, when the speech is attended to versus ignored. The distribution of the attentional boost across individuals is skewed above zero in all three EEG bands, consistent with positive attentional boost in the neural coding of target speech. Furthermore, there is considerable variation across subjects almost uniformly over the range of boosts. Finally, where a particular individual falls relative to the overall distribution is somewhat consistent across the three EEG bands (the rank correlation between the attentional boosts in the delta and theta bands is 0.78, and between the boosts in the delta and low-gamma bands is 0.38).

**Figure 7.**
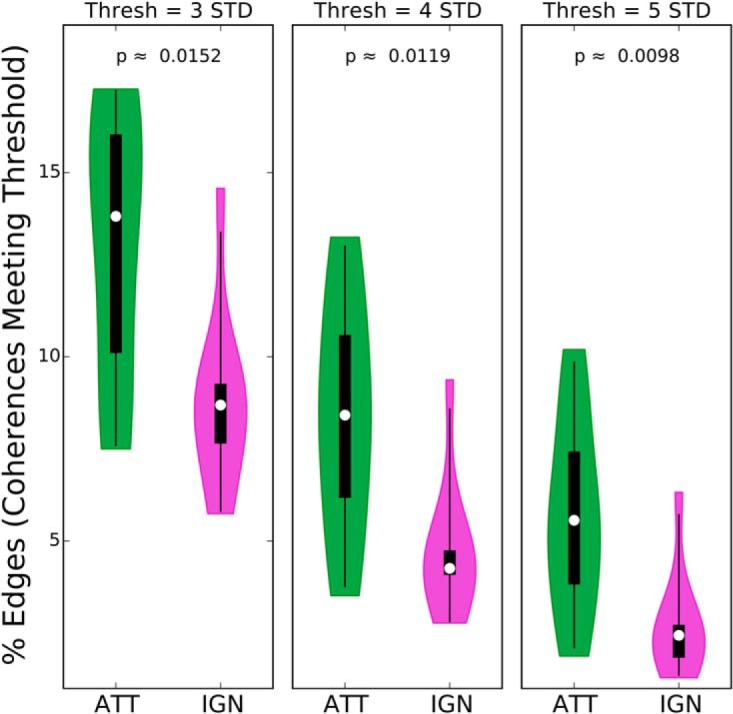
Percentage of edges (i.e., coherences meeting threshold) in attended (ATT) and ignored (IGN) speech-EEG bipartite graphs, at different coherence thresholds. The across-subject distribution of the percentage of graph edges is shown as a violin plot, separately for the attended and ignored conditions, and for three different coherence thresholds. In addition, the median (white dot), 50% confidence limits (thick black box), and 95% confidence limits (black whiskers) of each distribution are shown. Across all three threshold values, the number of edges is significantly larger for the attended condition (based on a permutation test; *p* values are shown). While Figure 3 showed that specific speech-EEG associations are strengthened by attention, the present result suggests that a greater number of distinct speech-EEG associations are induced by attention.

Although Figure 5 is visualized for a particular coherence threshold, the observation that there are many more edges in the attended condition than in the ignored condition did not depend strongly on the choice of threshold. To illustrate this, we quantified the percentage of edges (i.e., coherences that meet a given threshold) for the attended and ignored conditions, for three different threshold values. The results are shown in Figure 7. For all three thresholds shown, the number of edges in the attended condition is significantly greater than the number of edges in the ignored condition, which confirms the generality of this result. The *p* values for this statistical comparison were obtained using a permutation test as described in Materials and Methods, Statistical analysis. While Figure 3 showed that specific speech-EEG associations are strengthened by attention, the present result suggests that a greater number of distinct speech-EEG associations are induced by attention.

## Discussion

We systematically studied the attention-dependent relationships between input speech envelopes in different frequency bands and the neural response in different EEG channels and frequency bands. Importantly, we investigated selective attention effects in all canonical (Buzsáki and Draguhn, 2004) EEG frequency bands simultaneously. In doing so, we found that low-frequency delta-band and theta-band EEG showed the strongest attention effects (i.e., the greatest speech-EEG coherence increases for the attended condition compared to the ignored). This result is consistent with the preferential phase locking to attended rather than ignored speech in the delta and theta bands reported in previous EEG/MEG studies (Ding and Simon, 2012; O’Sullivan et al., 2015). Using stationary masking noise, Ding and Simon (2013) found that the delta band was the most robust in carrying target information at poorer SNRs (–3 dB and lower), whereas both delta and theta bands were equally robust in conveying target information at higher SNRs. These findings are consistent with our present results from using a speech masker at 0-dB SNR. One possible factor contributing to the strong delta-band and theta-band attention effects is that the power in the acoustic envelope of natural speech is maximal below 8 Hz (corresponding to the prosodic and syllabic rates; Ding et al., 2017). Moreover, in the presence of background noise, the SNR in the envelope domain at the auditory-nerve level is strongest for slow modulation frequencies (Rallapalli and Heinz, 2016). Thus, the strength of the delta- and theta-band effects may be a reflection of the neural computations that take advantage of the high power and SNR in speech at slow envelope frequencies. Yet another possible factor could be that attention mechanisms might be geared toward boosting the representation of those temporal modulations that are the most important for speech intelligibility; previous studies suggest that modulations below 8 Hz are perhaps the most important (Drullman et al., 1994; Elliott and Theunissen, 2009).

A novel finding of the present study is that the power fluctuations (i.e., envelope) of the low-gamma band of the EEG show significantly higher coherence with the attended speech stream versus the ignored. In contrast to cortical theta-band activity, activity in the gamma band has relatively small amplitude (Pritchard, 1992). This may explain why previous EEG studies have not reported attention effects in the gamma band. Despite the relatively low amplitude and the conservative statistical thresholding that we adopted (i.e., using Bonferroni corrections across EEG bands), we found the low-gamma envelope to fluctuate coherently with the attended speech. This finding supports the view that gamma activity plays an important role in the underlying physiologic computations that support selective listening (Tallon-Baudry and Bertrand, 1999; Ribary, 2005; Wang, 2010), and demonstrates that non-invasive EEG can be used to measure these effects.

While gamma-band responses have been investigated using EEG/MEG when processing speech streams in isolation, i.e., without competition (Gross et al., 2013), prior non-invasive studies of selective attention focused on the low-frequency portions of the brain signal, which overlap with traditional evoked responses (Luo and Poeppel, 2007; Ding and Simon, 2012; O’Sullivan et al., 2015). gamma-Band power has previously been shown to fluctuate coherently with the envelope of an attended speech stream in selective attention tasks, but only from invasive (ECoG) recordings (Mesgarani and Chang, 2012; Golumbic et al., 2013). The current results replicate this finding using EEG. However, one discrepancy in the gamma-band findings between the ECoG studies and the present EEG-based study is that the ECoG studies found the high-gamma, rather than the low-gamma band to be important, while we observed no significant effects at high-gamma. This may be explained by the fact that ECoG measurements are more spatially specific, reflecting local neural activity rather than the broadly distributed activity measured using EEG. For instance, the observed correlation of high-gamma in the spatially summed EEG signal with attended speech could be weak even if high-gamma activity within different brain areas are each significantly correlated with the speech, but at different phases. In general, the SNR of high-gamma signals measured from ECoG is likely greater than from EEG. The fact that we observed no significant attention-dependent change in the coherences between the high-gamma envelopes and speech signal envelopes is thus most likely due to limitations of scalp recordings.

One other study that examined the effect of attention on gamma-band EEG responses suggested that the attentional enhancement of gamma rhythms was specific to multisensory stimuli (audiovisual), and was not seen for stimuli presented solely to the auditory system (Senkowski et al., 2005); however, this study used simple tonal stimuli. Computational models (Börgers et al., 2008), *in vitro* studies (Llinas et al., 2002), *in vivo* electrophysiology (Fries et al., 2001), and modern studies using optogenetics (Cardin et al., 2009) show that gamma-band synchrony over a network of neurons can mediate sensory binding of different components that make up a perceptual object (Tallon-Baudry and Bertrand, 1999), which facilitates attentional selection and routing. Because the behavioral task in the current study involves both segregation (the grouping of input speech features into two separate coherent perceptual streams), and selection (the preferential, detailed processing of one of the two streams), the observed gamma-band effects could be related to either or both of those processes. Further studies are needed to understand the precise mechanisms involved in the generation of gamma-band activity, and how it shapes the network computations associated with segregation and selection (Shinn-Cunningham, 2008).

Despite the relatively high amplitude of the signals in the alpha and beta bands (e.g., compared to the gamma band), these mid-frequency bands did not show any attention effects. This is despite the fact that both the phase and envelope fluctuations of these bands were considered. At first glance, this result appears to be at odds with the findings of Obleser and colleagues (Obleser and Weisz, 2012; Wöstmann et al., 2016). However, the synchronous alpha variations in those studies were not of the overall alpha power, but rather the lateralization (i.e., left-right hemispherical asymmetry) of the alpha. Moreover, in Wöstmann et al. (2016), both the attended and ignored sound streams had the same temporal structure. This is in contrast to the present study, where the natural differences in the temporal envelope structure of distinct speech streams forms the basis of the analysis. Here, we did not examine any hemifield or hemisphere-specific aspects of attention on the EEG response. Instead, the goal was to examine the overall band-specific effects of attention on EEG responses. Analyses that focus on hemispheric lateralization of rhythms during spatial selective attention may indeed reveal alpha-band effects. Further, even for speech presented in isolation, cortical processing of linguistic sounds exhibits hemispheric asymmetry with a preferential left lateralization (Morillon et al., 2010). Future work should be undertaken to investigate hemifield-specific effects of attention on EEG, and how these effects interact with asymmetric aspects of cortical processing such as the left-lateralization of phonetic and linguistic processing.

On examining the scalp topography of the speech-EEG coherence, we found that the largest differences in coherence between the attended and ignored conditions occur in temporal-parietal channels, rather than EEG channels that are sensitive to early auditory responses. For example, the N100 EEG response, which is thought to originate from the primary auditory cortex, projects to Cz and Fz channels on the scalp. These channels show a weaker attention effect than the temporal-parietal channels, suggesting that early sensory responses are less modulated by attention than are later processing regions. This is consistent with the observation that attention effects can be localized to later “components” (200–220 ms) of the EEG response by methods such as spread-spectrum analysis, which allow for the temporal signature of the attention effect to be extracted (Power et al., 2012). These results suggest that higher-order processing areas selectively process attended speech.

In the present study, we also find individual differences in the overall magnitude of attentional enhancement of speech-EEG coherences, although all individuals scored >90% in the quiz. This finding is consistent with results from Choi et al. (2014), which used a selective attention task with complex-tone stimuli to show that there are large individual differences in the neural attentional boost, even when performance is at ceiling for all individuals. This study further found that as the behavioral demands became more adverse, the neural attentional boost from the easier condition was predictive of behavioral performance in the harder condition. Taken together with our results, this suggests that EEG measurements from an easier speech-based selective attention task may be used to quantify the top-down attentional contribution to individual differences in speech intelligibility in adverse listening conditions.

Finally, we visualized the coherences across all pairs of speech-EEG features as a bipartite graph, separately for each individual and for each attention condition. We found individual differences in the structures of attended and ignored graphs (i.e., which speech-EEG relationships were the strongest varied across individuals), and also in the set of EEG and speech features that are most informative about attentional focus in the entire network structure. Such an individual-specific set of just the most informative features can be used for individualized attention-decoding applications that require a compact feature set, such as attention-guided hearing aids (Fiedler et al., 2017; Fuglsang et al., 2017; O’Sullivan et al., 2017; Van Eyndhoven et al., 2017) and other BCIs. These features are likely to be more optimal for attention decoding than what may be extracted from more conventional analyses; however, the utility of this approach should be directly tested in future studies. One explanation for the individual differences reported here could be anatomic variations across people, which could lead to EEG measurements being differently sensitive across people to different sources. Another possibility is that every individual’s listening strategy might be different. For example, while some individuals may give more weight to spatial cues to perform the task, others may rely more on voice-based cues such as speaker pitch. Finally, there could also be individual differences in the efficacy of attentional modulation of different brain sources (Choi et al., 2014). To elucidate the precise reasons for the individual differences, future studies might consider using high-density recordings and source localization techniques.

## Synthesis

Reviewing Editor: Satu Palva, University of Helsinki Decisions are customarily a result of the Reviewing Editor and the peer reviewers coming together and discussing their recommendations until a consensus is reached. When revisions are invited, a fact-based synthesis statement explaining their decision and outlining what is needed to prepare a revision will be listed below. The following reviewer(s) agreed to reveal their identity: Hyojin Park. Note: If this manuscript was transferred from JNeurosci and a decision was made to accept the manuscript without peer review, a brief statement to this effect will instead be what is listed below.

Synthesis

In this manuscript, the authors describe their analysis of the neural correlates of attention during attention to one of two competing speech streams. The experiment appears to be well-designed and performed and the authors seem to understand. While there are not many novel findings, the results also confirm previous findings. It appears however that more detailed results might have been obtained from these data, and the description of the methods and presentation of the results are lacking. An improved version of the manuscript might be considered to sufficiently advance the field.

Title: it sounds too simplified. Neural representation, but it would be better if it could be more specific. Readers cannot guess from the title, e.g. if this is an fMRI study or an EEG spectral analysis.

Abstract: Instead of “coherence between each canonical EEG frequency band” it should say “the coherence between amplitude and phase of canonical frequency bands”, if I understood the methods correctly.

The expression “more features of the attended speech were represented more broadly” is unfortunate; firstly because the double use of “more” is confusing, secondly because “more broadly” is too vague.

Introduction

Line 102: Shouldn't it say “relationship between amplitude and phase of canonical EEG bands”?

Methods

Each story was heard as attended and ignored in different blocks. I was wondering if authors have checked if there was not any interference stemming from this design. For example, when the previously attended speech was presented as a to-be-ignored speech, if participants were having difficulty in ignoring it. Also in the opposite case where an ignored speech was presented as a to-be-attended speech, if participants somehow noticed it and if their attention was shifted. Both talks were normal (without noise-vocoded), so it would be possible that some words can be perceived and remembered even if it was instructed to be ignored. Line 155: It would be nice to have the mean and standard deviation for the number of epochs here.

Related to the point above, was distance of the presentation of the same talks adjusted (as attended and ignored), e.g. were the talks are at least one block separated?

How were the to-be-attended talks instructed? Was there a visual cue on the screen indicating a speech by female or male (by text or color etc.)?

How eye movement was instructed? Eyes closed or blinking naturally? If so, was there a fixation cross on the screen throughout the auditory attention?

Line 192ff: This is quite confusing. Why is “EEG itself” equated with the phase? Also, why say “envelope” and not “amplitude envelope”? (as this is what is meant, unless I misunderstood something) Finally, the mixing up of the terms “EEG bands” and “features” might cause some confusion with the readers.

Line 218ff: Over what frequencies did averaging take place? How many center frequencies were there in each frequency band? How were amplitude envelopes computed? This needs to be explained in more detail and clearer.

Line 236: What are, in brief, these criteria?

Line 248: The construction of graphs needs to be explained, however, see also my reservations about this analysis in general in the results section.

How would centrality measures be computed in a bipartite graph? These usually are applied to spatial graphs.

9. Page 11: Authors should mention the names of the open-source software (It sounds like MNE) in section 3.7 Software accessibility.

Results:

In general, the results presented in the manuscript are rather simplistic, given the complexity and richness of the data which decreases the novelty value of the findings and warrants their validity since not sufficient control data is shown. Figures 3-7 could be remade to convey more information and show additional data to convince the reader of the relevance of the findings.

Figure 3 now shows difference (“attended - ignored”) in coherence for each of the analysed EEG bands, but averaged across speech bands and EEG channels. This figure should also show the coherence between each EEG-band and with each speech-band in a second panel. Adding these data would yield information if different speech bands are correlated differentially with EEG data.

Also, why are only the average over all neuronal and all speech bands shown in Figure 4 and not the band specific correlations? One would assume that there are interesting results specific to certain speech bands and/or EEG bands as suggested by Figure 3. Figure 4 should show differential maps for each EEG band presented in Figure 3.

Authors also explain bilateral temporo-parietal regions for the difference between attended vs ignored speech, however, there is some hemispheric asymmetry that cannot simply described as “bilateral temporo-parietal regions”. Left side in the topo map looks more auditory-temporal located. Can a source analysis be done for to clarify which regions show more coherence with speech envelopes when attended?

Similarly, figure 5 should show coherence values separately for each frequency band not an average over bands as each narrow-band speech envelope conveys specific features in speech as interpreted as formant representations.

Figure 6 displays an unusual use of graph methodology and unusual visualization (usually graph analysis in neuroimaging is applied to spatial graphs). It is a unclear what is supposed to be the benefit of displaying the figure as is. These graphs only give a qualitative impression of the numbers of nodes and edges, but not a quantitative representation of their presence which should be the case. There is also no information in this figure about which bands and which electrodes have significant correlations. As of now, the information conveyed here is: “there are more edges in attended than ignored condition/stream”.

Alpha power modulation has been association with the attention effect in previous studies. Was attentional modulation of alpha power tested in the current study and can this effect be replicate, i.e. was the attentional allocation in the current study successful? Coherence values should be dependent by oscillatory power if they are not fully driven by the speech envelope.

Line 269: Shouldn't it be “stream” instead of “condition”?

Line 313: Please explain what a “formant” is, for those not familiar with the term.

Line 331: Again, problematic use of “more” twice in one sentence and of the vague term “more broadly”.

Figures

Figures should be in color not black and white.

Figure 7 should be a violin plots or similar showing also confidence limits.

## References

[B1] Bharadwaj HM (2018) SNAPsoftware/ANLffr: software tools for electrophysiology from the Systems Neuroscience of Auditory Perception Lab. Available at https://github.com/SNAPsoftware/ANLffr.

[B2] Bokil H, Purpura K, Schoffelen JM, Thomson D, Mitra P (2007) Comparing spectra and coherences for groups of unequal size. J Neurosci Meth 159:337–345. 10.1016/j.jneumeth.2006.07.011 16945422

[B3] Börgers C, Epstein S, Kopell NJ, (2008) Gamma oscillations mediate stimulus competition and attentional selection in a cortical network model. Proc Natl Acad Sci USA 105:18023–18028. 10.1073/pnas.0809511105 19004759 PMC2584712

[B4] Buzsáki G, Draguhn A (2004) Neuronal oscillations in cortical networks. Science 304:1926–1929. 15218136 10.1126/science.1099745

[B5] Cannon J, McCarthy MM, Lee S, Lee J, Börgers C, Whittington MA, Kopell N (2014) Neurosystems: brain rhythms and cognitive processing. Eur J Neurosci 39:705–719. 10.1111/ejn.12453 24329933 PMC4916881

[B6] Cardin JA, Carlén M, Meletis K, Knoblich U, Zhang F, Deisseroth K, Tsai L-H, Moore CI (2009) Driving fast-spiking cells induces gamma rhythm and controls sensory responses. Nature 459:663. 10.1038/nature08002 19396156 PMC3655711

[B7] Chermak GD, Musiek FE (1997) Central auditory processing disorders: new perspectives. San Diego: Singular Publishing Group 10.1055/s-0035-1564458

[B8] Cherry E (1953) Some experiments on the recognition of speech, with one and with two ears. J Acoust Soc Am 25:975–979. 10.1121/1.1907229

[B9] Choi I, Wang L, Bharadwaj H, Shinn-Cunningham B (2014) Individual differences in attentional modulation of cortical responses correlate with selective attention performance. Hear Res 314:10–19. 10.1016/j.heares.2014.04.008 24821552 PMC4096237

[B10] Ding N, Simon JZ (2012) Emergence of neural encoding of auditory objects while listening to competing speakers. Proc Natl Acad Sci USA 109:11854–11859. 10.1073/pnas.1205381109 22753470 PMC3406818

[B11] Ding N, Simon JZ (2013) Adaptive temporal encoding leads to a background-insensitive cortical representation of speech. J Neurosci 33:5728–5735. 10.1523/JNEUROSCI.5297-12.2013 23536086 PMC3643795

[B12] Ding N, Patel AD, Chen L, Butler H, Luo C, Poeppel D (2017) Temporal modulations in speech and music. Neurosci Biobehav Rev 81:181–187. 10.1016/j.neubiorev.2017.02.011 28212857

[B13] Dobie RA, Wilson MJ (1989) Analysis of auditory evoked potentials by magnitude-squared coherence. Ear Hear 10:2–13. 10.1097/00003446-198902000-00002 2721825

[B14] Dobie RA, Wilson MJ (1994) Objective detection of 40 Hz auditory evoked potentials: phase coherence vs. magnitude-squared coherence. Electroencephalogr Clin Neurophysiol 92:405–413. 10.1016/0168-5597(94)90017-5 7523084

[B15] Doelling KB, Arnal LH, Ghitza O, Poeppel D (2014) Acoustic landmarks drive delta–theta oscillations to enable speech comprehension by facilitating perceptual parsing. Neuroimage 85:761–768. 10.1016/j.neuroimage.2013.06.035 23791839 PMC3839250

[B16] Drullman R, Festen JM, Plomp R (1994) Effect of temporal envelope smearing on speech reception. J Acoust Soc Am 95:1053–1064. 10.1121/1.408467 8132899

[B17] Elliott TM, Theunissen FE (2009) The modulation transfer function for speech intelligibility. PLoS Comput Biol 5:e1000302. 10.1371/journal.pcbi.1000302 19266016 PMC2639724

[B18] Engel AK, Fries P (2010) Beta-band oscillations--signalling the status quo? Curr Opin Neurobiol 20:156–165. 10.1016/j.conb.2010.02.015 20359884

[B19] Faust K (1997) Centrality in affiliation networks. Soc Networks 19:157–191. 10.1016/S0378-8733(96)00300-0

[B20] Fiedler L, Wöstmann M, Graversen C, Brandmeyer A, Lunner T, Obleser J (2017) Single-channel in-ear-eeg detects the focus of auditory attention to concurrent tone streams and mixed speech. J Neural Eng 14:036020. 10.1088/1741-2552/aa66dd 28384124

[B21] Fries P, Reynolds JH, Rorie AE, Desimone R (2001) Modulation of oscillatory neuronal synchronization by selective visual attention. Science 291:1560–1563. 10.1126/science.1055465 11222864

[B22] Fuglsang SA, Dau T, Hjortkjær J (2017) Noise-robust cortical tracking of attended speech in real-world acoustic scenes. Neuroimage 156:435–444. 10.1016/j.neuroimage.2017.04.026 28412441

[B23] Ghitza O, Greenberg S (2009) On the possible role of brain rhythms in speech perception: intelligibility of time-compressed speech with periodic and aperiodic insertions of silence. Phonetica 66:113–126. 10.1159/000208934 19390234

[B24] Ghitza O, Giraud AL, Poeppel D (2012) Neuronal oscillations and speech perception: critical-band temporal envelopes are the essence. Front Human Neurosci 6:340. 10.3389/fnhum.2012.00340 PMC3539830 23316150

[B25] Giraud AL, Poeppel D (2012) Cortical oscillations and speech processing: emerging computational principles and operations. Nat Neurosci 15:511–517. 10.1038/nn.3063 22426255 PMC4461038

[B26] Goh KI, Choi IG (2012) Exploring the human diseasome: the human disease network. Brief Funct Genomics 11:533–542. 23063808 10.1093/bfgp/els032

[B27] Gold B, Morgan N (2002) Vocoders In: Speech and audio signal processing: processing and perception of speech and music, pp 431–447. Singapore: Wiley.

[B28] Golumbic EMZ, Ding N, Bickel S, Lakatos P, Schevon CA, McKhann GM, Goodman RR, Emerson R, Mehta AD, Simon JZ, Poeppel D, Schroeder CE (2013) Mechanisms underlying selective neuronal tracking of attended speech at a “cocktail party.” Neuron 77:980–991. 10.1016/j.neuron.2012.12.037 23473326 PMC3891478

[B29] Gramfort A, Luessi M, Larson E, Engemann DA, Strohmeier D, Brodbeck C, Goj R, Jas M, Brooks T, Parkkonen L, Hämäläinen M (2013) MEG and EG data analysis with MNE-Python. Front Neurosci 7:267. 10.3389/fnins.2013.00267 24431986 PMC3872725

[B30] Gramfort A, Luessi M, Larson E, Engemann DA, Strohmeier D, Brodbeck C, Parkkonen L, Hämäläinen MS (2014) Mne software for processing meg and eeg data. Neuroimage 86:446–460. 10.1016/j.neuroimage.2013.10.027 24161808 PMC3930851

[B31] Greenwood DD (1990) A cochlear frequency-position function for several species--29 years later. J Acoust Soc Am 87:2592–2605. 10.1121/1.399052 2373794

[B32] Gross J, Hoogenboom N, Thut G, Schyns P, Panzeri S, Belin P, Garrod S (2013) Speech rhythms and multiplexed oscillatory sensory coding in the human brain. PLoS Biol 11:e1001752 10.1371/journal.pbio.1001752 24391472 PMC3876971

[B33] Hannan EJ (1970). Inference about spectra In: Multiple time series, Vol 1, pp 245–324. Hoboken, NJ: Wiley.

[B34] Khanna SM, Leonard DG (1982) Basilar membrane tuning in the cat cochlea. Science 215:305–306. 10.1126/science.7053580 7053580

[B35] Kolaczyk ED, Csárdi G (2014) Statistical analysis of network data with R, Vol 65 New York: Springer.

[B36] Kryter KD (1962) Methods for the calculation and use of the articulation index. J Acoust Soc Am 34:1689–1697. 10.1121/1.1909094

[B37] Kumar G, Amen F, Roy D (2007) Normal hearing tests: is a further appointment really necessary? J R Soc Med 100:66. 10.1177/014107680710000212 17277271 PMC1791002

[B38] Lachaux J, Rodriguez E, Martinerie J, Varela F (1999) Measuring phase synchrony in brain signals. Hum Brain Mapp 8:194–208. 10619414 10.1002/(SICI)1097-0193(1999)8:4<194::AID-HBM4>3.0.CO;2-C PMC6873296

[B39] Lin FR, Niparko JK, Ferrucci L (2011) Hearing loss prevalence in the United States. Arch Intern Med 171:1851–1853. 10.1001/archinternmed.2011.506 22083573 PMC3564588

[B40] Llinas RR, Leznik E, Urbano FJ (2002) Temporal binding via cortical coincidence detection of specific and nonspecific thalamocortical inputs: a voltage-dependent dye-imaging study in mouse brain slices. Proc Natl Acad Sci USA 99:449–454. 10.1073/pnas.012604899 11773628 PMC117580

[B41] Loizou PC (2013) Speech enhancement: theory and practice, part III: evaluation, Ed 2 Boca Raton: CRC Press.

[B42] Luo H, Poeppel D (2007) Phase patterns of neuronal responses reliably discriminate speech in human auditory cortex. Neuron 54:1001–1010. 10.1016/j.neuron.2007.06.004 17582338 PMC2703451

[B43] Mesgarani N, Chang EF (2012) Selective cortical representation of attended speaker in multi-talker speech perception. Nature 485:233–236. 10.1038/nature11020 22522927 PMC3870007

[B44] Morillon B, Lehongre K, Frackowiak RS, Ducorps A, Kleinschmidt A, Poeppel D, Giraud A-L (2010) Neurophysiological origin of human brain asymmetry for speech and language. Proc Natl Acad Sci USA 107:18688–18693. 10.1073/pnas.1007189107 20956297 PMC2972980

[B45] Nichols TE, Holmes AP (2002) Nonparametric permutation tests for functional neuroimaging: a primer with examples. Hum Brain Mapp 15:1–25. 11747097 10.1002/hbm.1058 PMC6871862

[B46] Obleser J, Weisz N (2012) Suppressed alpha oscillations predict intelligibility of speech and its acoustic details. Cereb Cortex 22:2466–2477. 10.1093/cercor/bhr325 22100354 PMC4705336

[B47] O’Sullivan JA, Power AJ, Mesgarani N, Rajaram S, Foxe JJ, Shinn-Cunningham BG, Slaney M, Shamma SA, Lalor EC (2015) Attentional selection in a cocktail party environment can be decoded from single-trial EEG. Cereb Cortex 25:1697–1706. 10.1093/cercor/bht355 24429136 PMC4481604

[B48] O’Sullivan J, Chen Z, Herrero J, McKhann GM, Sheth SA, Mehta AD, Mesgarani N (2017) Neural decoding of attentional selection in multi-speaker environments without access to clean sources. J Neural Eng 14:056001. 10.1088/1741-2552/aa7ab4 28776506 PMC5805380

[B49] Power AJ, Foxe JJ, Forde E-J, Reilly RB, Lalor EC (2012) At what time is the cocktail party? a late locus of selective attention to natural speech. Eur J Neurosci 35:1497–1503. 10.1111/j.1460-9568.2012.08060.x 22462504

[B50] Pritchard WS (1992) The brain in fractal time: 1/f-like power spectrum scaling of the human electroencephalogram. Int J Neurosci 66:119–129. 10.3109/00207459208999796 1304564

[B51] Rallapalli VH, Heinz MG (2016) Neural spike-train analyses of the speech-based envelope power spectrum model: application to predicting individual differences with sensorineural hearing loss. Trends Hear 20:2331216516667319 10.1177/2331216516667319

[B52] Ribary U (2005) Dynamics of thalamo-cortical network oscillations and human perception. Prog Brain Res 150:127–142. 10.1016/S0079-6123(05)50010-4 16186020

[B53] Senkowski D, Talsma D, Herrmann CS, Woldorff MG (2005) Multisensory processing and oscillatory gamma responses: effects of spatial selective attention. Exp Brain Res 166:411–426. 10.1007/s00221-005-2381-z 16151775

[B54] Shannon RV, Zeng F-G, Kamath V, Wygonski J, Ekelid M (1995) Speech recognition with primarily temporal cues. Science 270:303–304. 10.1126/science.270.5234.303 7569981

[B55] Shinn-Cunningham B (2008) Object-based auditory and visual attention. Trends Cogn Sci 12:182–186. 10.1016/j.tics.2008.02.003 18396091 PMC2699558

[B56] Slaney M (1993) An efficient implementation of the Patterson-Holdsworth auditory filter bank. Apple Comp Tech Rep 35 Available at https://engineering.purdue.edu/~malcolm/apple/tr35/PattersonsEar.pdf.

[B57] Slepian D (1978) Prolate spheroidal wave functions, Fourier analysis, and uncertainty V: the discrete case. Bell Syst Tech J 57:1371–1430. 10.1002/j.1538-7305.1978.tb02104.x

[B58] Smith ZM, Delgutte B, Oxenham AJ (2002) Chimaeric sounds reveal dichotomies in auditory perception. Nature 416:87–90. 10.1038/416087a 11882898 PMC2268248

[B59] Tallon-Baudry C, Bertrand O (1999) Oscillatory gamma activity in humans and its role in object representation. Trends Cogn Sci 3:151–162. 10.1016/S1364-6613(99)01299-1 10322469

[B60] Thomson D (1982) Spectrum estimation and harmonic analysis. Proc IEEE 70:1055–1096. 10.1109/PROC.1982.12433

[B61] Uusitalo MA, Ilmoniemi RJ (1997) Signal-space projection method for separating meg or eeg into components. Med Biol Eng Comput 35:135–140. 10.1007/bf02534144 9136207

[B62] Van Eyndhoven S, Francart T, Bertrand A (2017) Eeg-informed attended speaker extraction from recorded speech mixtures with application in neuro-steered hearing prostheses. IEEE Trans Biomed Eng 64:1045–1056. 10.1109/TBME.2016.2587382 27392339

[B63] Wang XJ (2010) Neurophysiological and computational principles of cortical rhythms in cognition. Physiol Rev 90:1195–1268. 10.1152/physrev.00035.2008 20664082 PMC2923921

[B64] White JA, Banks MI, Pearce RA, Kopell NJ (2000) Networks of interneurons with fast and slow *γ*-aminobutyric acid type a (gabaa) kinetics provide substrate for mixed gamma-theta rhythm. Proc Natl Acad Sci USA 97:8128–8133. 10.1073/pnas.100124097 10869419 PMC16681

[B65] Wilson TP (1982) Relational networks: an extension of sociometric concepts. Soc Networks 4:105–116. 10.1016/0378-8733(82)90028-4

[B66] Wöstmann M, Herrmann B, Maess B, Obleser J (2016) Spatiotemporal dynamics of auditory attention synchronize with speech. Proc Natl Acad Sci USA 113:3873–3878. 10.1073/pnas.1523357113 27001861 PMC4833226

